# Genetic determination of the ovarian reserve: a literature review

**DOI:** 10.1186/s13048-021-00850-9

**Published:** 2021-08-06

**Authors:** Aleksandra V. Moiseeva, Varvara A. Kudryavtseva, Vladimir N. Nikolenko, Marine M. Gevorgyan, Ara L. Unanyan, Anastassia A. Bakhmet, Mikhail Y. Sinelnikov

**Affiliations:** 1 Sechenov University, Mohovaya 11c10, Moscow, Russian Federation; 2 Moscow State University, Moscow, Russian Federation; 3 Saratov State Medical University, Moscow, Russian Federation; 4 Research Institute of Human Morphology, Moscow, Russian Federation

## Abstract

The ovarian reserve is one of the most important indicators of female fertility. It allows for the evaluation of the number of viable oocytes. This parameter is actively used in pregnancy planning and in assisted reproductive technology application, as it determines chances of successful fertilization and healthy pregnancy. Due to increased attention towards diagnostic tests evaluating the ovarian reserve, there has been a growing interest in factors that influence the state of the ovarian reserve. True reasons for pathological changes in the ovarian reserve and volume have not yet been explored in depth, and current diagnostic screening methods often fall short in efficacy. In the following review we analyze existing data relating to the study of the ovarian reserve through genetic testing, determining specific characteristics of the ovarian reserve through genetic profiling. We explore existing studies dedicated to finding specific genetic targets influencing the state of the ovarian reserve.

## Introduction

“Ovarian reserve” is a term that is used to describe the remaining capacity of oocytes in the ovary. With age, the ovarian reserve tends to naturally diminish and normally does not present with pathological changes. Environmental impact, physiological, hormonal, iatrogenic and other factors determine the state of the ovarian reserve. Recently, genetic defects have been directly associated with a significant reduction in the ovarian reserve [1]. In cases of infertility, it is now recommended to perform genetic screening [2, 3] in order to assess the extent and management possibilities of existing defects.

Genetic profiles FMR1, EIF4ENIF1, BRCA1/2, H19, HMGB2, ADR-α1, 2, ADR-β2, NR5A1, ATG7, ATG9A, KHDRBS1, FIGLA, 22q11.2, SPO11, HFM1, GDF9, TP53 have been shown to play a key role in ovarian reserve determination [4, 5]. Presently, studies have established several important factors that influence the basal ovarian volume and appropriate ovarian response after ovarian hyperstimulation during in-vitro fertilization (IVF) treatment. However, many genetically determined characteristics are not sufficiently explored and require further evaluation. In this review we aim to define specific genetic profiles and factors that predispose to early decline of the ovarian reserve.

## Materials and methods

The PubMed, Scopus, Web of Science and eLibrary databases were searched using the following key words and their combination: “ovarian reserve”, “ovarian reserve AND genetics”, “ovarian reserve AND gen”, “ovarian reserve AND genetic”, “ovarian reserve AND epigenetic NOT cancer”, “ovarian reserve AND genetic screening” and “diminished ovarian reserve”. Original studies were included in this review. Meta-analyses and systematic reviews were screened for references applicable to our search criteria.

## Results

**Fig. 1:**
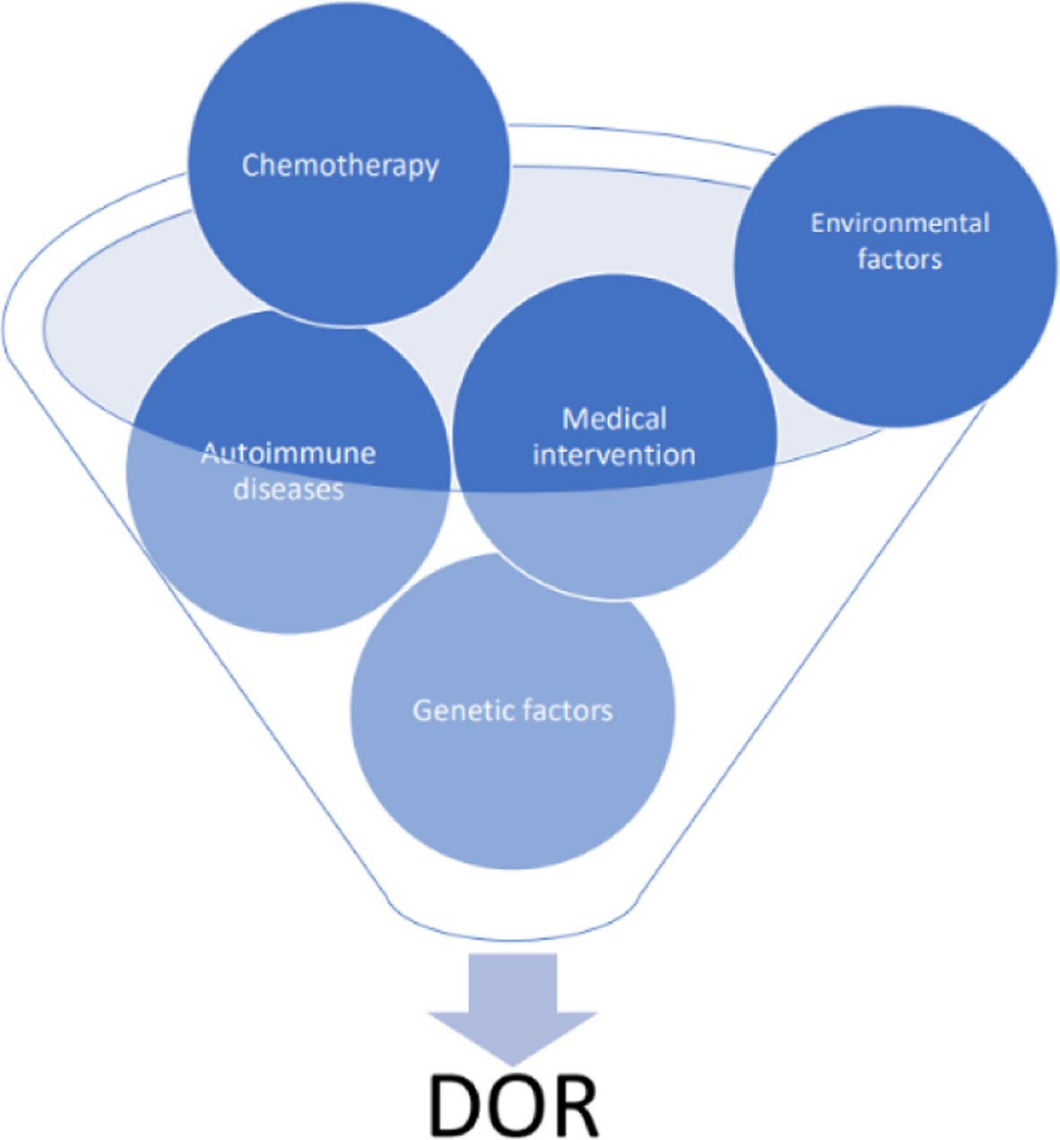
Factors affecting the ovarian reserve, leading to diminishment of the ovarian reserve

Low ovarian reserve is an increasing social problem, as many women are at high risk for early decline of the ovarian reserve. A low reserve is one of the leading causes of female infertility and up to 26% of women who are undergoing fertility treatment with assisted reproductive technology (ART) have a diminished ovarian reserve (DOR) [6]. Infertility, in turn, leads to various psychological and physical disorders [7]. Diagnostic evaluation of the etiology of ovarian decline is complex, and is aimed at finding individual factors, including genetic and epigenetic, that may have caused early DOR. Besides genetic factors, autoimmune, gynecological, systemic diseases, iatrogenic manipulations (surgery), chemotherapy and environmental impact have been shown to play a significant role in reduced ovarian reserve (Fig. 1) [5]. These factors must be taken into account when using assisted reproductive technologies during in-vitro fertilization.

Current data is unanimous that polycystic ovary syndrome (PCOS) is characterized by an increase in the level of sex hormones. This underlined the importance of dose reduction of administered gonadotropins in order to avoid ovarian hyperstimulation [8]. In obese patients, studies show that more gonadotropic hormones stimulation is required to achieve a sustainable therapeutic effect [9]. In addition to changing the dosage of drugs, for various conditions the approach to the IVF procedure can be adjusted. For example, research shows the efficacy of myo-inositol (MI) and D-chiro-inositol (DCI) (insulin-sensitizing agents) in polycystic ovary syndrome [10]. In the case of endometriosis, it is recommended to use IVF as a secondary treatment for women who cannot achieve pregnancy following laparoscopic surgery for up to 12 months [11].

In this review, we delineate several categories of genetic factors according to their influence on the ovarian reserve (OR).

### Fragile X Mental Retardation Genes (FMR)

Fragile X Mental Retardation Genes (*FMR*) are a family of regulator genes located in the X chromosome. The Fragile X Mental Retardation 1 (*FMR1*) gene is commonly associated with neuropsychiatric disorders, but it has been shown to play a role in other pathologies as well. Apart from classical *FMR1* disorder symptoms (tremor and ataxia), ovarian dysfunction is also common [12]. *FMR1* mutations provoke different pathological changes along with the decline of the ovarian reserve.

**Fig. 2:**
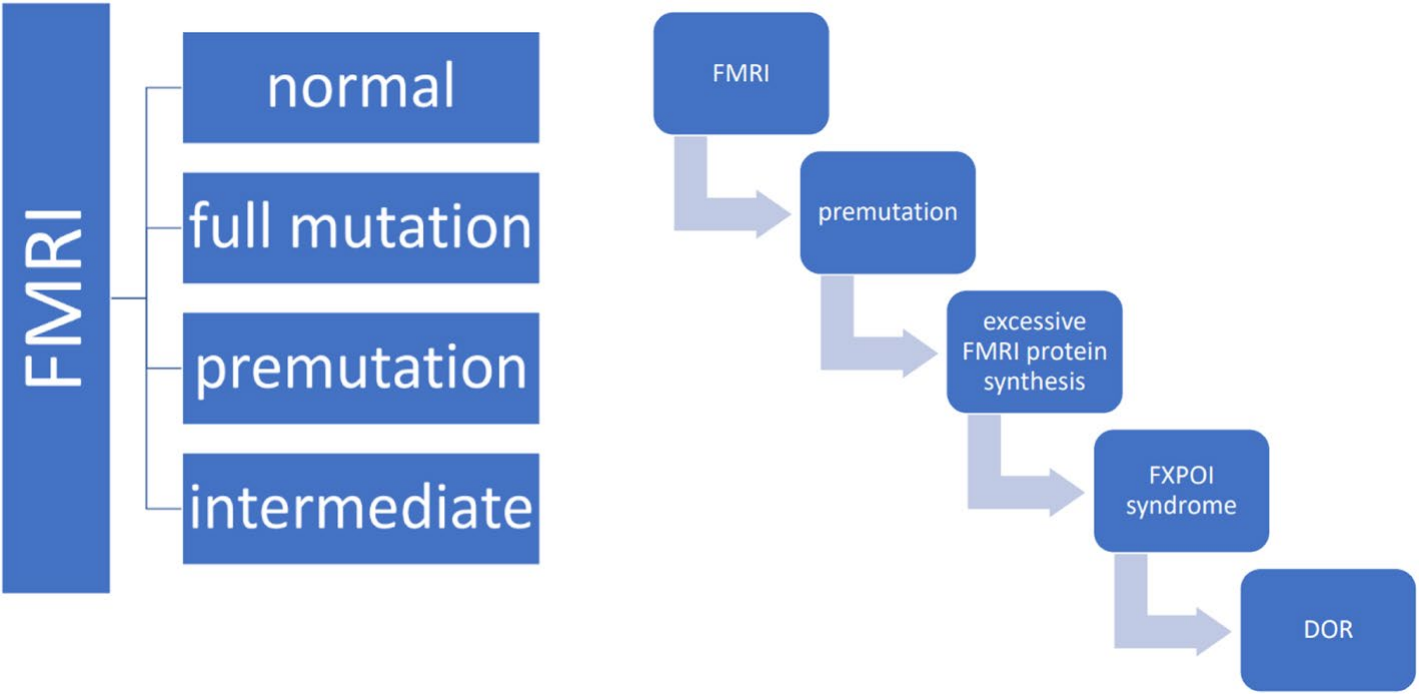
Alleles of the FMRI gene and their role in DOR

The *FMR1* gene contains a 5’-UTR triplet repeat (GGG) region, the length of which varies individually. It has been shown that *FMR1* gene expression depends on the length and number of these triplet repeats [13]. Four allelic forms of the *FMR1* gene are identified according to the number of GGG repeats (Fig. 2): normal (< 45 CGG); full mutation (> 200 CGG), which influences complete gene suppression; premutation (55–200 CGG), which causes excessive synthesis of FMR1 in cells; intermediate mutant allele (45–55 CGG) [14]. The premutation allelic form manifests in two conditions: Fragile X-associated tremor/ataxia syndrome (FXTAS), associated with X chromosome suppression; and Fragile X-associated primary ovarian insufficiency (FXPOI) syndrome. The later was identified in 1990s and was a pilot study showing direct relationship between genetic pathological changes and low ovarian volume [15].

Approximately 20% of women with permutation have FXPOI and significant decline of ovarian reserve [12]. Studies show that the rate of permutation is 1:150–300 in the female population but it this constant varies significantly dependent on ethnicity, race, environmental and other factors [16, 17]. GGG repeats are normally interrupted by intermediate AGG repeats which are essential for stabilization of the genetic sequence. Abnormal interruption of GGG repeats has been shown to influence the formation of the ovarian reserve through impact on the *FMR1* gene transcriptional activity [18]. As such, due to various mutations of the FMR1 gene, preimplantation diagnostic screening is recommended to identify permutation [19].

### Single-nucleotide polymorphisms (SNPs)

**Fig. 3:**
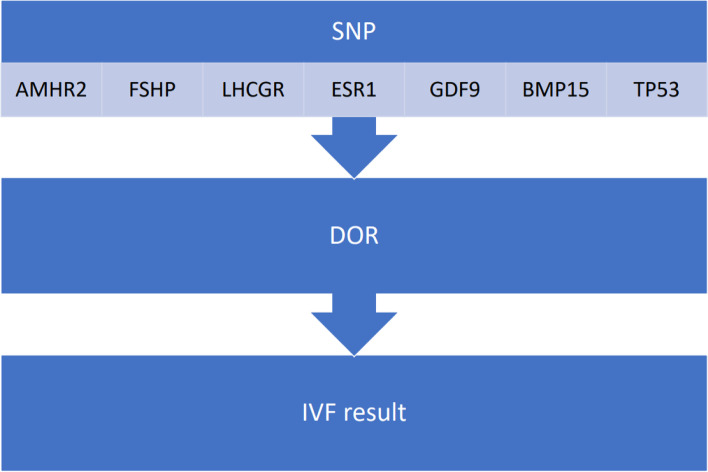
The impact of SNPs on reproductive health

Single-nucleotide polymorphisms are another form of genetic variability. They are caused by point mutations, so they exist in high abundance in human genome. They are found in genes coding the receptors of the Anti-Müllerian hormone – AMH (AMHR2), Follicle-stimulating hormone – FSH (FSHR), luteinizing hormone – LH (LHCGR), estrogen (ESR), growth and differentiation factors (*GDF9*). They are also found in genes that are responsible for bone morphogenetic proteins – BMPs (gene *TR53*) [20, 21]. SNPs have been shown to play an important role in poor ovarian response (POR) and DOR development (Fig. 3). POR characterizes the response of the ovaries to hormonal stimulation and is directly related to DOR, but these concepts are distinguished. The concept of POR is used in reproductive medicine and includes an insufficient response of the ovaries to the introduction of large doses (more than 300 IU / day) of gonadotropins, when in the stimulation regimens used in IVF programs it is not possible to ensure the growth and maturation of more than 3 follicles. In POR, the selection of oocytes and embryos is not based on an indicator of their quality, but only on the characteristics of viability, which reduces the effectiveness of treatment.

SNPs of FSH receptor are considered to be potential diagnostic genetic markers of POR [21], as FSH plays an crucial role in the formation of ovarian follicles, since after binding to the receptor, this hormone triggers many signaling reactions and other activating molecular mechanisms in cells. It is therefore regarded as a key marker of ovarian reserve status. The FSH receptor is a transmembrane protein in granulosa cells [22]. It’s activation causes granulosa cell proliferation. It was shown that single-nucleotide polymorphisms in loci associated with FSH (680, 307, 189), exon 10, are responsible for diminished ovarian reserve [22–24]. Due to the fact that FSH receptor plays a dominant role in the regulation of folliculogenesis, SNPs such as rs6165 and rs6166 are have been shown to correlate with changes in the ovarian reserve [25, 26]. SNPs 29G > A and 919G > A in the FSH receptor are associated with POR, by causing its inactivation and halting folliculogenesis [27]. AMH also has a prominent role in folliculogenesis regulation and ovarian. AMH receptor SNPs polymorphisms, such as AMHRII-482A > G (23,24) have been shown to increase the risk of DOR [28–30]. LH, a key regulator of ovarian response to stimulus, is essential in regulation of the ovarian reserve. SNPs in the LH receptor, including rs4539842 (a set of six base pairs CTGCAG), rs12470652 (c.827A > G/p.Asn 291Ser), and rs2293275 (c.935G > A/p.Ser312Asn) impact the ovarian reserve and promote early POR [31, 32].

The *ESR1* gene is located in the 6^th^ chromosome and is responsible for coding the ESR protein, which plays an important role in regulating granulosa cell proliferation and folliculogenesis. SNPs of gene *ESR1* have been shown to cause POR, but existing data is contradictive. Some research shows a direct correlation between *ESR1* SNPs and POR [33], but other research indicates no obvious correlation [34]. This discrepancy may be due to the fact that POR is a multivariable condition, and *ESR1* SNPs alone may not contribute entirely to POR.

Polymorphisms in genes *GDF9, C398G, C447T, BMP15* have been shown to negatively impact ovarian response among females who are subject to controlled ovarian hyperstimulation. Existing data shows that *GDF9* polymerphisms plays an important role in different stages of folliculogenesis [35]. *GDF9* and *BMP15* belong to a superfamily of transforming growth factors (beta) [36]. The SNPs of genes *GDF9* and *BMP15* have been identified: c.-9C > G (rs3810682), BMP15:c.328 + 905A > G (rs3897937), BMP15:c.852C > T (rs17003221); GDF9:c.134-694G > A (rs4705974), GDF9:c.-31-951G > A (rs11748063), GDF9:c.-152G > C (rs30177), GDF9:g.1073C > T (rs803224) [36, 37]. These polymorphisms negatively influence folliculogenesis. The BMP15:c.852C > T SNP has been identified as a factor of DOR and ovarian reserve genetic marker [36]. Polymorphism of gene *TR53* has also been shown to impact the state of the ovarian reserve and consequently the results of in vitro fertilization [38].

### Non-coding RNA molecules

miRNA and piRNA have been shown to have an impact on granulosa cells with low ovarian volume because of RNA interference (RNAi) and various epigenetic rearrangements which determine genetic expression, making them an important target in genetic diagnostics and targeting in DOR [39].

The impact of miRNA-106a on the pathogenesis of DOR is actively studied. Its’ action results in the lowering of the viability of granulosa cells and stimulate apoptosis through activation of apoptosis signal-regulating kinase 1 (ASK1) [40]. Additionally, miRNA-23a has been shown to participate in the development of folliculogenesis disorders and, as a result, DOR [41].

**Fig. 4:**
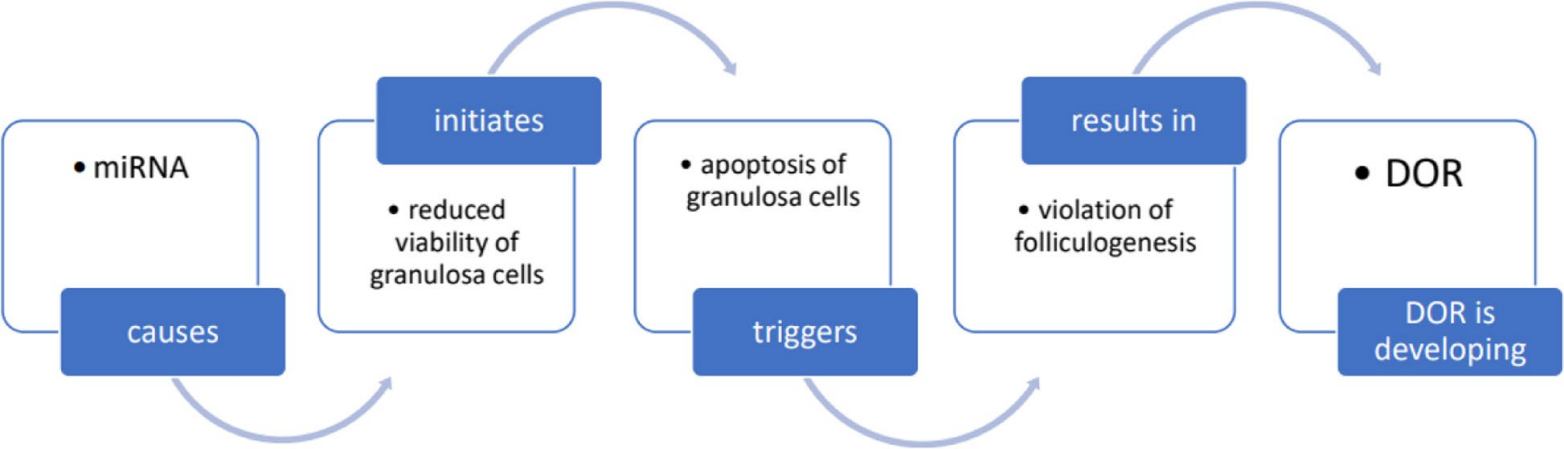
The effect of non-coding miRNAs on the ovarian reserve

The decline of long non-coding RNA H19 (part of the conserved imprinted gene cluster that predetermines fetal stages of development) in the blood serum has been shown to be connected with the decline of AMH and an increased risk of extreme POR, therefore acting as a potential biomarker of POR and DOR [42]. Further studies are required to evaluate the full extent of non-coding miRNA role in ovarian role regulation, as existing data underlines their importance (Fig. 4).

### Genes involved in formation of primordial and pre-antral follicles

Biallelic mutations of Folliculogenesis Specific BHLH Transcription Factor (FIGLA) gene (*FIGLA)* (specifically 2 T > Cp.Met1Thr), have been shown to cause a decline in the ovarian reserve [43]. The mentioned mutation does not determine gene transcription but blocks whole protein synthesis of FILGA which plays an important role in the formation of primordial follicles. Biallelic recessive mutations are connected to a loss of function of FIGLA, have been known to cause DOR [44]. Analysis of genome sequencing showed that mutations of *ATG7* and *ATG9A* genes (connected with programmed cell death) cause reduced biosynthesis of autophagosomes (causing defects in autophagy, the mechanism regulating primary follicle differentiation) which may lead to DOR through folliculogenesis dysregulation [45]. Research showed the key role of Janus-kinase 1 (genetic profile *JAK1*) in regulating activation of primary follicles and supporting the ovarian reserve. A range of integrated inner pathways, including JAK-STAT, have been shown to be responsible for regulation of the ovarian reserve, primary follicle growth and female fertility [46]. As such, disruption of JAK1 and it’s pathways in associated genetic profiles is of interest in assessment of the ovarian reserve. Keratin gene mutations, resulting in it’s pathology has been shown to affect ovarian health through immunohistochemical evaluation of K8/K18 expression. Changes in K8/K18 expression in the ovaries are associated with increased depletion of the ovarian reserve, which leads to primary ovarian insufficiency [47]. No specific genetic profiles have been studied in the case of Keratin structure and ovarian reserve health.

### Factors regulating gene expression and epigenetic changes

**Fig. 5:**
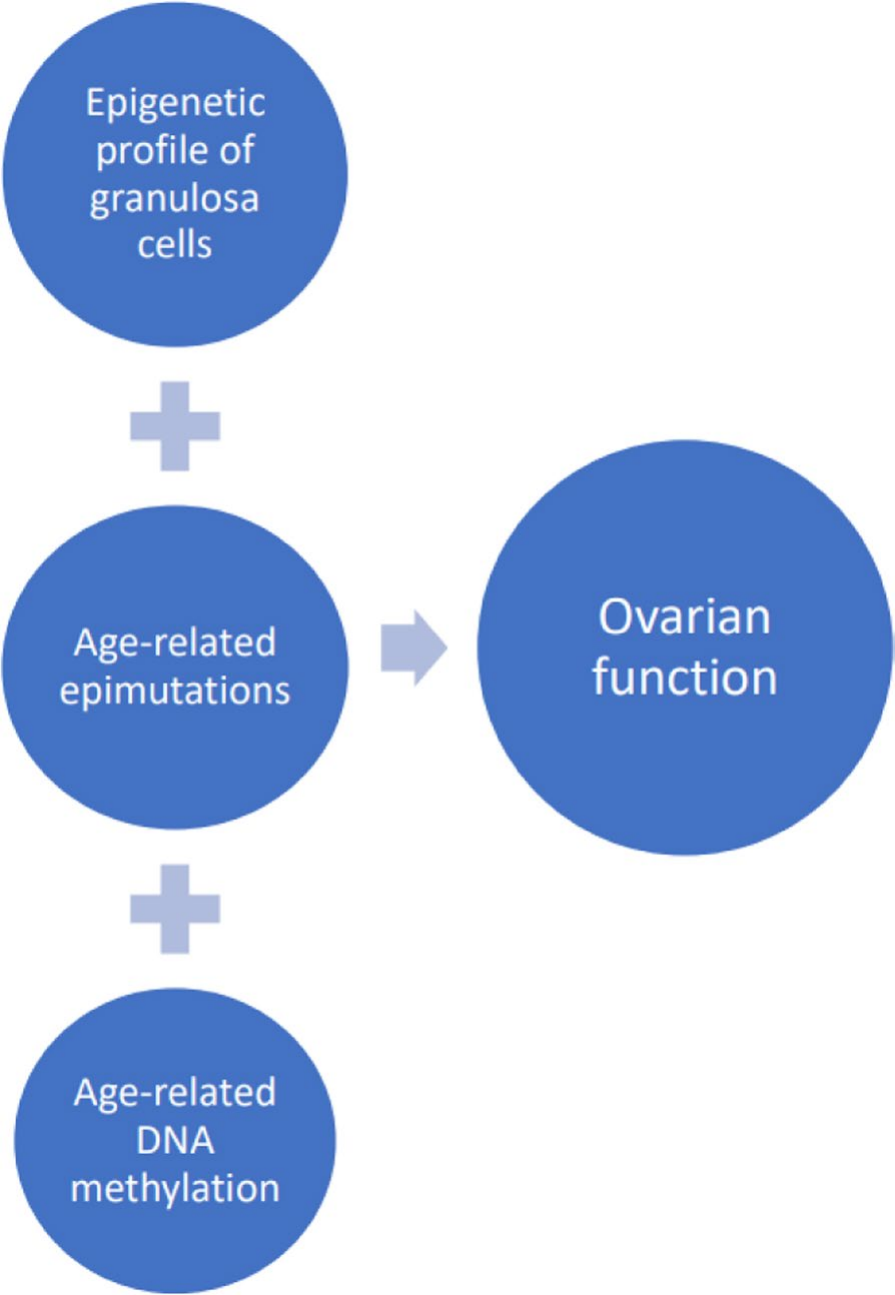
Epigenetic factors that affect the functioning of the ovaries

Besides the previously mentioned factors, specific signaling proteins involved in metabolic regulation (sirtuins) have been shown to play a critical role in ovarian pathogenesis. Thus, a range of changes in sirtuin genetic profiles such as *SIRT1*, *SIRT2*, *SIRT4*, *SIRT5*, *SIRT6*, *SIRT7*, which regulate epigenetic gene silencing and suppress recombination of rDNA, can lead to pathological changes in the ovaries. Accumulation of epimutations associated with sirtuin disfunction, age-related abundance of methylated regions in ovarian DNA have a considerable impact on their functions (Fig. 5) [48].

Interestingly, among women with low ovarian volume the influence of ADR-α1, 2 and ADR-β2 are predetermined by the depletion of gene expression and this has been shown to result in ovarian aging and DOR [49].

### Genes responsible for germ cell formation, meiosis, DNA repair

Recent studies show that *BRCA* mutations cause a decline of the ovarian reserve and premature ovarian aging, accumulation of mutations in the DNA of female germ cells and primary follicle atresia. *BRCA1/2* are responsible for homologous recombination of DNA and influence DNA repair (double-strand break – DSB), which are caused by ataxia–telangiectasia (AT). BRCA and AT DNA repair have been shown to play an important role the process of early onset ovarian dysfunction. *BRCA1/2* and AT-related DSB and DNA repair tend to weaken with age in different oocytes [50]. The number of primary follicles decreases and an increased accumulation of double-strand DNA breaks in oocytes is seen. Women with *BRCA1/2* mutations have low ovarian volumes and early menopause [50]. Existing data hints that BRCA1/2 mutations may play an important role in ovarian reserve dysregulation.

Whole exome sequencing allowed to identify a new heterozygous missense mutation in the HFM-1 (human homologue of yeast Mer3) gene (*HFM1*). This pathological mutation is associated with DOR and causes defects of RNA splicing [51]. The defects of an alternative splicing are considered to be potential mechanisms which cause pathological decline of the ovarian reserve [51]. According to existing data, a heterozygous variation of *KHDRBS1* is one of the reasons of such pathological changes. They appear in the form of delayed puberty and considerable depletion of secondary and pre-antral follicles. Thus, a more detailed examination of mutation will help to understand etiology, a molecular mechanism of pathological changes in the ovarian reserve [52, 53].

### Genes that have a somatic effect

It is important to understand that the reviewed genes are not specific to the reproductive system, but also have a complex effect on the body, as most genetic profiles do. *FMR1, SIRT, BRCA, BMP15, TP53, ADR, KHDPBS* have varible expression profiles and therefore variable effects. For example, a premutation in the *FMR1* gene is associated with Fragile X-associated tremor/ataxia syndrome (FXTAS), which manifests itself as a progressive neurodegenerative disease with pronounced manifestations. tremor, ataxia, dementia, behavioral changes, and much more. Clinical manifestations include cerebellar ataxia, action tremor, parkinsonism, cognitive impairment, psychiatric disorders, peripheral neuropathy, and autonomic dysfunction of varying in severity, are possible. In women, due to the presence of a second X chromosome, this syndrome does not develop, however, it is known that carriers are more likely to suffer from depression and anxiety disorders. They also have an increased risk of developing primary ovarian failure [54]. This association is important to understand the varying profile of effect and expression on targeted genes. Additionally it is worth noting that mutations in the *SIRT* gene family, as well as other epigenetic disorders, have multiple aggravating consequences, including premature aging, neurodegenerative diseases, cancer, oxidative stress, and autophagy [55].

*BRCA* genetic expression products are known regulators of cellular repair and lifecycle, maintaining the stability of the genome. *BRCA1*/*2* mutations lead to disturbances in the mechanisms of molecular repair and cell division. Carriers have an increased risk of cancers of the breast, ovaries, fallopian tubes, peritoneum, prostate, pancreas, stomach, gallbladder and bile ducts, and melanoma. If the mutation is inherited from both parents, Fanconi anemia, malignant tumors and acute myeloid leukemia may develop [56].

Bone morphogenetic protein (BMP), a product of *BMP* gene cluster expression, is a growth factor and morphogenetic signaling protein family that is involved in structural organization of tissues. BMPs act on cells through specific BMP receptors (BMPRs) and play an important role in the development of the heart, central nervous system, and skeleton. Impaired BMR signaling can have pathologic effects on a developing embryo. Mutations in *BMP* and BMP inhibitors cause a number of diseases [57].

The p53 protein is an expression product of the *TP53* gene, a transcription factor that regulates the cell cycle. p53 functions as a tumor suppressor and the *TP53* gene is regarded as an anti-oncogene. Numerous studies have shown that imbalances in the expression of p53 isoforms and mutations in the TP53 gene cause such debilitating disorders as cancer, premature aging and degenerative diseases [58].

The *ADR* cluster genes are involved in the regulation of lipid and carbohydrate metabolism, blood pressure, and heart function through expression products—adrenergic receptors. Mutations and polymorphisms of these genes can lead to the development of various diseases: arterial hypertension, heart disorders, ischemia, obesity, type I and II diabetes mellitus and insulin resistance [59, 60].

The protein product of *KHDPBS* gene expression belongs to the STAR (signal transduction and activation of RNA) family and regulates the splicing of target genes, which plays an important role in the formation of contacts and myelination in the nervous system. STAR products are also involved in the embryonic development of the nervous system. Disorders of *KHDPBS* genes are associated with diseases such as schizophrenia and autism, as well as with other neurodegenerative pathologies [61].

### New findings on genes

**Fig. 6:**
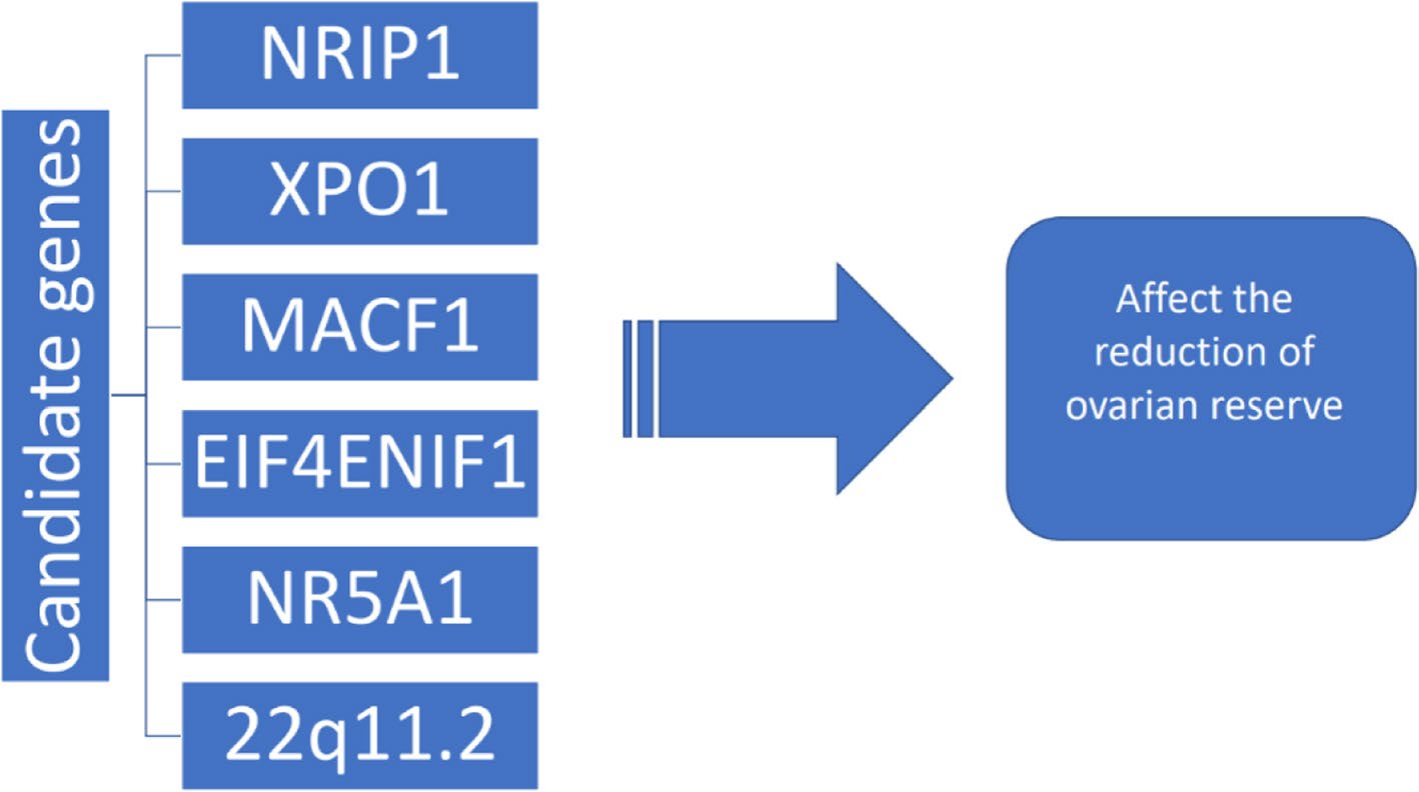
New genes with a potential role in ovarian reserve pathology and homeostasis

Recent studies have identified new candidate genes which may be involved in ovarian pathogenesis (Fig. 6). These include *NRIP1*, *XPO1* and *MACF1*, which have been shown to be related to ovarian functioning, but their role in the human body is not fully understood [62]. A new gene mutation *EIF4ENIF1* has been discovered recently, and is identified in patients with low ovarian volume, prompting investigation and increased interest to the role of genetic factors in ovarian homeostasis [63]. New variations of gene *NR5A1* have been shown to influence the decline of the ovarian reserve, causing primary ovarian insufficiency, which can cause primary ovarian insufficiency (POI) and infertility [64]. Besides, 22q11.2 changes are identified among women with low ovarian volume and POI [65].

## Conclusions

Genetic factors should be taken into account in the treatment and diagnosis of ovarian reserve pathology. Identifying genetic predisposition to early ovarian reserve depletion may serve beneficial in family planning. Existing data show an abundant amount of research on genetic and epigenetic profiles which may influence ovarian reserve formation and consistency. More so, several specific genetic markers have been identified to be associated with ovarian reserve depletion through several important pathological pathways. Further research is needed to evaluate the unsupported conclusions and unproven correlations between genetic mutations and ovarian reserve stability. Inconsistent data shows that the ovarian reserve is subjected to a multifactorial influence, and disruption of separate factors may not consistently lead to ovarian reserve pathology.

## Data Availability

All data and materials are available from the corresponding author upon reasonable request.
